# *Lejeunea hodgsoniana*, a newly described, long recognised *Lejeunea* (Jungermanniopsida, Lejeuneaceae) from lowland coastal forest habitats in New Zealand

**DOI:** 10.3897/phytokeys.29.5376

**Published:** 2013-11-11

**Authors:** Rodney J. Lewington, Peter Beveridge, Matt A. M. Renner

**Affiliations:** 1 4 Highbury Crescent, Highbury, Wellington 6012, New Zealand; 2 Museum of New Zealand Te Papa Tongarewa, PO Box 465, Wellington, New Zealand; 3 Royal Botanic Gardens and Domain Trust, Mrs Macquaries Road, Sydney, NSW 2000, Australia

**Keywords:** *Lejeunea hodgsoniana*, Lejeuneaceae, Jungermanniopsida, New Zealand, new species

## Abstract

*Lejeunea hodgsoniana* Grolle ex R.J.Lewington, P.Beveridge & M.A.M.Renner **sp. nov.**, A taxon originally recognised by Riclef Grolle in 1980, but not described, known from a number of coastal sites in the North Island, the northern extremity of the South Island, the Kermadec Islands, and the Chatham Islands of New Zealand, is described and illustrated. The species is distinctive amongst species of *Lejeunea* in the Australasian flora in the combination of complanate shoots, relatively large broadly-ovate leaf lobes, with some lobules bearing prominent multicellular triangular teeth on a base of two to four cells, the flattened perianths having a faint dorsal carina. Its publication brings the number of species recognized for New Zealand to 14, seven of which are currently considered endemic.

## Introduction

The Museum of New Zealand Te Papa Tongarewa herbarium (WELT) holds five *Lejeunea* specimens bearing determinavit slips signed by the late Riclef Grolle, dated 1980 and carrying the name *Lejeunea hodgsoniana*. The specimens had been collected in 1969–1970 by B.G. Hamlin, botanist and curator of the herbarium, at that time in the National Museum, later incorporated into Te Papa. They were loaned to Grolle, with other *Lejeunea* specimens, when he was preparing his study of the Lejeuneaceae in Tasmania (Grolle 1982). Grolle may have had the intention to describe this species. It appears, however, that the name was not published and we are unaware of any available description. In the meantime, it has become practice to refer to the species by the tag name *Lejeunea ‘hodgsoniana’* Grolle ined. e.g. (Renner et al. 2010). Apart from androecia in one of the packets, the WELT collections are sterile. Recently-collected fertile material has been selected as the basis for an overdue formal description of this taxon. It is apparent that, in naming the specimens *Lejeunea hodgsoniana*, Grolle wished to recognise the contribution made to the study of New Zealand liverworts by Mrs E.A. Hodgson, 1888–1983, who during the period 1941–1972, had published a series of papers on a wide range of genera, including *Schistochila* (Hodgson 1941), *Heteroscyphus* (Hodgson 1943) and *Radula* (Hodgson 1944), but not on any member of the Lejeuneaceae (Godley 1984). Grolle had previously honoured this contribution in his naming of *Solenostoma hodgsoniae* (Grolle) J.J. Engel and *Lepidogyna hodgsoniae* (Grolle) R.M. Schust.

Most Lejeuneaceae genera in New Zealand, including *Lejeunea*, have never been the subject of comprehensive regional revision. At best they have been either studied opportunistically in response to discovery of new entities (Grolle 1973, Glenny 1996, Renner 2010, Renner and Pocs 2011, Renner and de Lange 2011, Renner et al. 2009) or included as part of studies of other regions (e.g. Grolle 1982). New Zealand appears to have a large endemic component to its Lejeuneaceae flora (Renner 2010) and this, in combination with the lack of recent study, means the family is poorly known (Renner and Pocs 2011) and several new entities await description.

Here we formally describe the longest known of these new species, Grolle’s unpublished *Lejeunea ‘hodgsoniana’*, and are pleased to publish the name on his behalf. Its publication brings the number of species recognized for New Zealand to 14 (based on Glenny 1998 with recent additions by Renner et al. 2010 and Renner and de Lange 2011), seven of which are currently considered endemic. Some of these fourteen species are known to be complexes requiring further investigation (i.e. *Lejeunea epiphylla* Colenso non Mitt. auct. and *Lejeunea primordialis* Taylor). Despite this, the higher than average rate of endemism for *Lejeunea* mirrors that of *Austrolejeunea*, another well-studied genus of Lejeuneaceae, and again suggests the existence of a distinct, even if not particularly diverse, southern-temperate Australasian element within this otherwise predominantly tropical family.

## Taxonomic treatment

### Lejeunea hodgsoniana

Grolle ex R.J.Lewington, P.Beveridge et M.A.M.Renner sp. nov.

http://species-id.net/wiki/Lejeunea_hodgsoniana

#### Diagnosis.

Differs from other antipodal Lejeunea species in its relatively large leaf and shoot size, the presence of a multicellular first lobule tooth on a base of two to four cells, combined with complanate shoots, distant elliptic-ovate underleaves with deep sinus and narrow lobes that are usually capped by a single pointed apical cell, and an obcordate perianth with a broad flattened dorsal surface before inflation, and reduced dorsal carina.

#### Type.

**NEW ZEALAND. Porirua**: Titahi Bay, Stuart Park, track to cliff edge at S end of bay: Sounds-Wellington Ecological Region, Wellington Ecological District, on trunk and branches of *Melicytus ramiflorus* in coastal thicket with *Pittosporum crassifolium* and *Coprosma repens*. Bryophyte associates, *Cololejeunea minutissima*, *Frullania monocera*, *Frullania patula*, *Lejeunea colensoana*, *Rhynchostegium muriculatum*, *Siphonolejeunea nudipes* and *Syntrichia papillosa*, 41°06'33"S, 174°49'43"E, ca. 15m, 19 July 2012, P. Beveridge MB-2. (Holotype: WELT [WELT H012563], isotypes AK, CHR, F, NSW).

Plants bright green, not pellucid, grey-green in herbaria, forming conspicuous more or less circular mats to 7.0 cm diameter, or more extensive mats by confluent growth. (Figure 3) Shoots 1.0–1.5 mm wide, ca. 12 mm long. Branching of the *Lejeunea*-type frequent, shoots occasionally exhibiting more or less pinnate growth patterns but more often forming diffuse complanate wefts by continued lejeuneoid branching.

**Figure 1.**
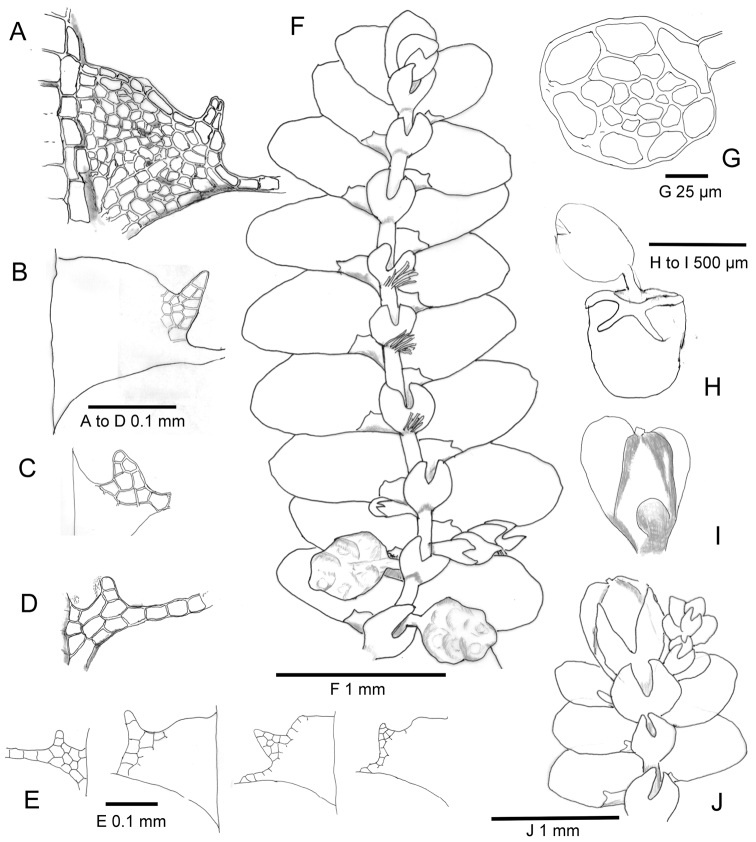
*Lejeunea hodgsoniana* – Morphological features. **A** and **B** Well-developed lobule **C–E** Lobules showing the variety of forms from the same stem **F** Shoot with androecia **G** Stem cross-section **H** Inflated perianth with emergent sporophyte **I** Perianth before enlargement of the sporophyte showing the lateral and ventral carinae **J** Leading shoot showing a terminal gynoecium and a subfloral innovation. (All from type.) **A–D**: lobules scale bar 0.1 mm, **E**: four lobules, scale bar 0.1 m, **F**: scale bar 1mm, **G**: scale bar 25 µm, **H–I**: scale bar is 0.5 mm and **J** scale bar 1 mm.

**Figure 2.**
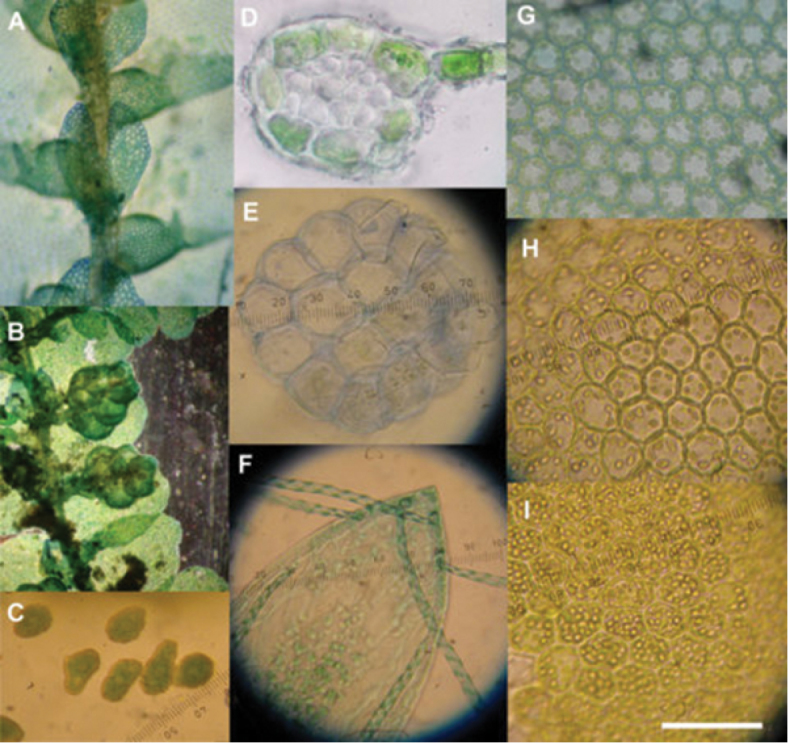
*Lejeunea hodgsoniana*. **A** Shoot showing the underleaf with a deep narrow sinus and long narrow lobes, and well-developed lobules **B** Androecia **C** Spores **D** Stem cross-section **E** Seta cross-section **F** Apices of a sporophyte valve showing elaters and pseudoelaters **G–I** Leaf cells showing the variation in oil body density. (**G** stained).

Stem (Figures 1G and 2D) 90–125 µm in diameter with 7 cortical cells, walls 2–3 µm thick, and with ca.12 rows of smaller medullary cells. Ventral merophyte of two rows with cells sub-quadrate to rectangular, 22–45 µm × 22–30µm. Lateral merophytes with shared mid-dorsal row, the alternate contribution of each lateral merophyte to the row, 5–6 (8) cells long, with cells quadrate to rectangular, 22–45 µm × 17–24 µm, the contribution boundaries marked by oblique cross walls, by the antical lobe insertion of the contributing merophyte encroaching weakly onto the mid-dorsal row and by the position of a papilla.

**Figure 3.**
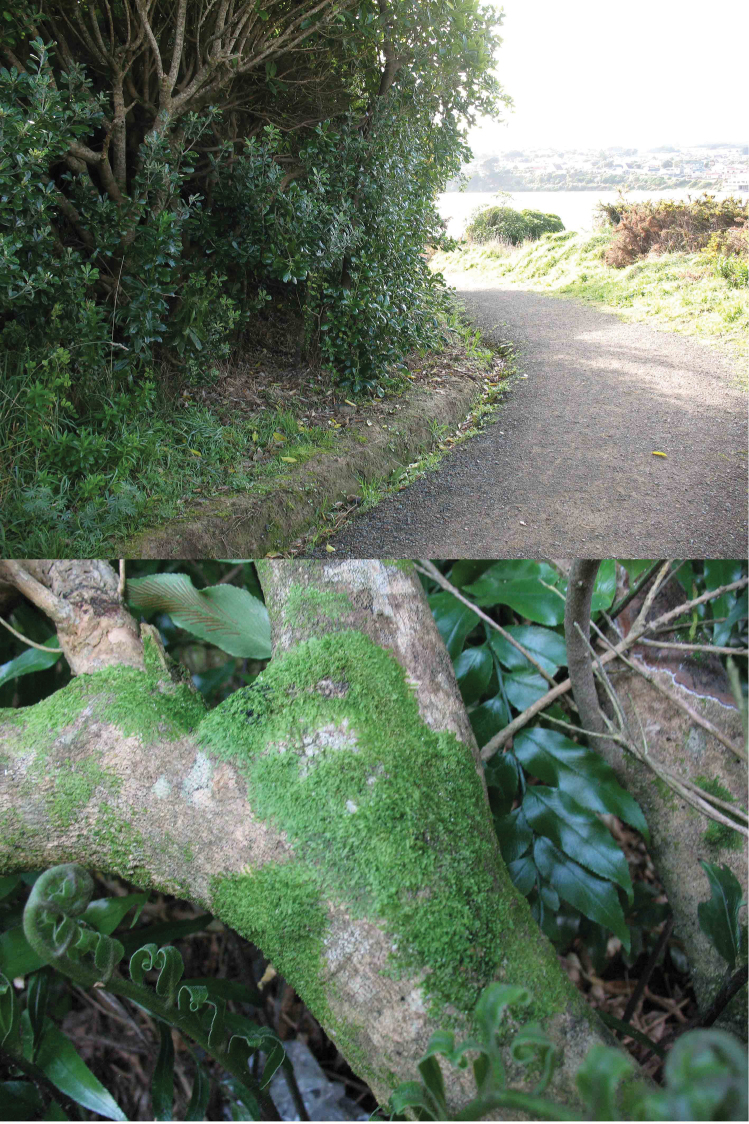
Type locality. Above. Regenerating native coastal bush. Below. Trunk of Melicytus ramiflorus showing the extensive mats of Lejeunea hodgsoniana resulting from confluent growth.

Leaves (Figure 1F) alternate, incubous, shortly imbricate or contiguous, leaf lobes broadly ovate, on leading shoots 750–950 µm long by 550–650 µm wide, on branches 300–700 µm long × 200–600 µm wide, complanate in arrangement along the shoot and more or less flat when fresh, flat or weakly convex above when dry, at 75°–90°to the stem, margins entire, more or less straight or slightly crenulate by weakly bulging cells. Apex rounded, occasionally obtusely pointed, antical lobe margin at insertion evenly rounded or variably ampliate, projecting onto or across the stem. Postical leaf margin decurrent. Insertion 0.5 lobe width, sub-longitudinal. Two-celled leaf-free stem gutter. Lobe mid-laminal cells, (Figure 2G) hexagonal, isodiametric or slightly elongate, 17.5 × 17.5 µm to 25 × 30 µm, basal lamina cells similar, occasionally in longitudinal rows, isolated cells to 37.5 µm long, marginal cells rectangular 12.5–20 × 12.5–20 µm. Cells thin-walled in young leaves, trigones absent or weakly developed. Walls to 2 µm in older leaves. Cuticle smooth.

Mid laminal cell oil bodies in younger leaves (Figure 2G and H) (2) 4–6 (7), spherical 4–6 µm, less commonly ellipsoidal or fusiform 6–7.5 (10) µm × 4–5 (6) µm, pale grey to light brown, coarse granular to sub-botryoidal. Oil bodies in older leaves near gametangia, occasionally 14–20, then densely filling the cell (Figure 2I).

Lobules (Figure 1A–E) polymorphic and relatively small, 0.023–0.125 of lobe area, best developed towards the apex of leading shoots, there 90–240 µm at insertion with a 100–300 µm carina, the free margin weakly inflated, in-rolled or not, arcuate or more or less straight, bearing a multicellular sub-triangular apical tooth, two to four cells wide at the base, up to 15 cells in total, arched or straight, usually pointing towards the stem apex. Lobule papilla proximal to the tooth and a further papilla at the free margin-stem axil. More distant from the shoot apex the lobules are explanate, the tooth diminishing to three cells on a base of two cells, to two uniseriate cells, or to a single cell. Amongst and postical to the gametangia, the lobules are usually more uniformly small explanate triangular, insertion ca. 50 µm, with a single-celled tooth, carina ca. 50 µm.

Underleaves (Figures 1F and 2A) usually distant, contiguous or imbricate only at the stem apex, appressed to stem, elliptic-ovate, widest below mid leaf, in leading shoots 320–350 µm long × 260–300 µm wide, smaller to 200 × 170 µm in branch shoots, sinus 0.5–0.6, narrow U or occasionally V-shaped. Attachment transverse, occasionally to two, typically to three cortical cells. Where three, to the lateral merophyte row bearing the adjacent lobule, as well as to the two ventral merophyte rows, with or without a pair of enlarged basal marginal cells. Lobes 6–9 cells wide at base, usually ending in a single cell, occasionally two uniseriate, very occasionally two cells juxtaposed.

Rhizoids, where present, 100–200 µm long, hyaline, in fascicles arising from a cluster of basal underleaf cells forming a disk of ca. 25 rhizoid initials.

Asexual propagules not seen.

Autoicous. Androecia (Figure 1F and 2B) ca. 500 µm long × 500 µm wide, usually on short determinate achlorophyllose lateral branches, often obscured from above by the shoot leaves, occasionally terminal on short leafy lateral shoots. One (2) proximal underleaves often connate on one side with a proximal small sterile bract. Fertile male bracts 2–4 pairs, becoming smaller distally, each bearing one or two spherical antheridia, each ca. 100 µm diameter, supported by a filament of uniseriate cells.

Gynoecia (Figure 1H–J) occasionally terminal on leading shoots, subtending a single subfloral lejeuneoid innovation to continue shoot growth, more often terminal on lateral branches which may be repeated in continued lejeuneoid shoot sequences.

Female bract lobes connate, sub-symmetrical or asymmetrical, obovate-spathulate to lingulate, apices rounded or obtuse to broadly acute, spreading to squarrose, 450–750 µm long, 180–450 µm wide. Lobule ligulate, 220–450 µm long, 50–80 µm wide, erect or arching inwards from broad or narrow sinus, 0.5–0.7. A common asymmetry has a broad obovate-spathulate female bract with rounded apex and a short arched lobule paired with a narrow lingulate bract with acute apex and long erect lobule. Bracteole 300–600 µm long, 220–300 µm wide, lobes usually erect, occasionally spreading, 5–7 cells wide at the base, often with a small lateral shoulder on each outer lobe margin, sinus U shaped, usually narrow, occasionally broad, to 0.6, lobes ending in two uniseriate cells, or a single cell. Bracteole unequally biconnate to female bract bases.

Perianths (Figure 1H and I) before distortion by enlargement of the sporophyte, dorso-ventrally compressed, pentacarinate, with well-developed lateral and ventral carinae and a reduced dorsal carina. Perianth obcordate in profile, broadest below the broadly rounded apices of the lateral carinae, 500–750 µm high by ca. 80 µm wide at the base, ca. 500 µm wide at the apex, the ventral carinae before inflation as two conspicuous oblique plicae converging on the plane ventral surface below the rostrum, a short dorsal carina ca. 88 µm long as an obscure low-profile ridge immediately below the rostrum on the broad plane dorsal perianth surface, sometimes wanting. Rostrum 45 µm wide, 37.5–50 µm, two to three cells high, positioned in a variably-expressed depression between spreading apices of the lateral carinae. Estipitate.

Sporophyte capsule (Figure 1H) spherical, light brown at maturity, ca. 300 µm in diameter. Tiered seta (Figure 2E) to 1.5 mm long, usually only shortly emergent from the perianth, 150 µm wide, in cross section, 16 rows of thin-walled hyaline cells, 12 cortical and four medullary, cells isodiametric, ca. 37.5 µm wide, ca. 80 µm long. Capsule dehiscent into four erect, incompletely separated valves, the valve sinus 0.75 the valve length (Figure 2F).

Cells of the valve outer layer differentiated into three distinct areas. Firstly, in the apical part of the valve, a marginal layer of quadrate to rectangular cells with firm walls, some with a single nodular thickening on median walls or broader sheet thickening, joined by a single row of rhomboidal cells with similar thickening to a conspicuous median cluster of about 12 large, relatively thin-walled elongate-hexagonal cells without wall thickening, the largest four, ca. 62.5 × 32.5 µm. Secondly, a median basal cluster of thin-walled quadrate cells. Thirdly above the junction of adjacent valves on each side, small quadrate marginal cells with conspicuous sheet thickening on the medial walls bordering a cluster of three to four rows of rhomboidal cells with sheet and nodular thickening and variably sinuose walls. Below the valve junction a row of four large quadrate-trapezoid cells extending to the hypophysis basal cell, together with the row of the adjacent valve, forming a conspicuous triangular group.

Cells of the capsule inner layer quadrate at the apex margin, otherwise rhomboidal, longer than outer layer cells with rounded ends, elliptical near valve junction, quadrate at the base.

Inner layer inner tangential walls with hyaline to light brown ornamentation in two valve regions. At the valve apex, bell and discoid thickening present at the points of attachment of the elaters and weakly extending onto adjacent cells along cell boundaries. A more extensive area of ornamentation at mid-valve with a dense array of bell and discoid thickening along axes with a more or less longitudinal orientation, not clearly related to cell boundaries, here the thickenings flare slightly onto the inner radial cell wall. The precise relationship of the thickening to inner layer cell walls could not be resolved.

Elaters and pseudoelaters on familiar pattern of 5 (2) and 4 (2), with similar opposite valve pairs, two bearing five elaters, one attached at the valve apex and two each side close to the valve apex, the other valve pairs (Figure 2F) lacking the apical elater, all valves with two pseudoelaters attached by their length to the valve inner layer. Elaters with weak unihelical thickening.

Spores, (Figure 2C) light brown ellipsoids, symmetrical or asymmetric, finely and densely papillose, 32.5–37.5 × 17.5–20 µm. Precocious germination not seen.

#### Distribution and habitat.

*Lejeunea hodgsoniana* is known from a number of locations in New Zealand ranging from latitude 29°14'39"S in the Kermadec Islands to latitude 44°20'S on Pitt Island in the Chatham Islands. In the northern half of the North Island, it is recorded from off-shore islands on the eastern coast from Poor Knights Island, south through the islands of the outer and inner Hauraki Gulf including the Mokohinau Islands, Hen and Chicken Group, Little Barrier Island and The Noises, and from the Mercury Islands Group and Mayor Island east of the Coromandel Peninsula. There are also a small number of northern mainland collections from North Cape south to Port Waikato and Hamilton. In the southern North Island, locations are mainly coastal in the vicinity of Wellington, including on Mana Island. On the South Island it is known from a single collection from the base of Farewell Spit. Elevation is generally less than 100 m with the altitudinal range from 1m to about 520 m, the latter in the Kermadec Islands.

Four of the collections are from shaded stream bed rock, serpentinite at North Cape, basalt or basaltic andesite elsewhere. In most of its locations, however, *Lejeunea hodgsoniana* has been corticolous in coastal forest or scrub. Species of *Melicytus*, *Melicytus* aff. *ramiflorus* in the Kermadecs, *Melicytus chathamicus* in the Chatham Islands, and *Melicytus ramiflorus* elsewhere are the most frequently cited phorophytes or associates. Other cited phorophytes are: *Acacia dealbata*, *Agathis australis*, *Beilschmiedia tarairi*, *Brachyglottis repanda*, *Coprosma macrocarpa*, *Coprosma repens*, *Cordyline obtecta*, *Geniostoma ligustrifolium*, *Hoheria populnea*, *Kunzea* spp., *Meryta sinclairii*, *Metrosideros excelsa*, *Metrosideros kermadecensis*, *Myrsine divaricate*, *Olearia traversiorum*, *Pittosporum umbellatum*, *Rhopalostylis sapida*, *Streblus banksii*, *Vitex lucens*, and apple tree (*Malus x domestica*).

Bryophyte associates have included *Archilejeunea olivacea*, *Codonoblepharon minutus*, *Cololejeunea minutissima*, *Fabronia australis*, *Racopilum* sp., *Frullania monocera*, *Frullania patula*, *Frullania pentapleura*, *Frullania rostellata*. *Lejeunea colensoana*, *Lejeunea helmsiana*, *Lejeunea oracola*, *Lejeunea primordialis*, *Lepidolaena taylorii*, *Lopholejeunea colensoi*, *Metalejeunea cucullata*, *Metzgeria furcata*, *Neckera hymenodonta*, *Rhynchostegium muriculatum*, *Siphonolejeunea nudipes*, *Syntrichia papillosa*, *Tetraphidopsis pusilla* and *Thuidium sparsum*.

**Figure 4.**
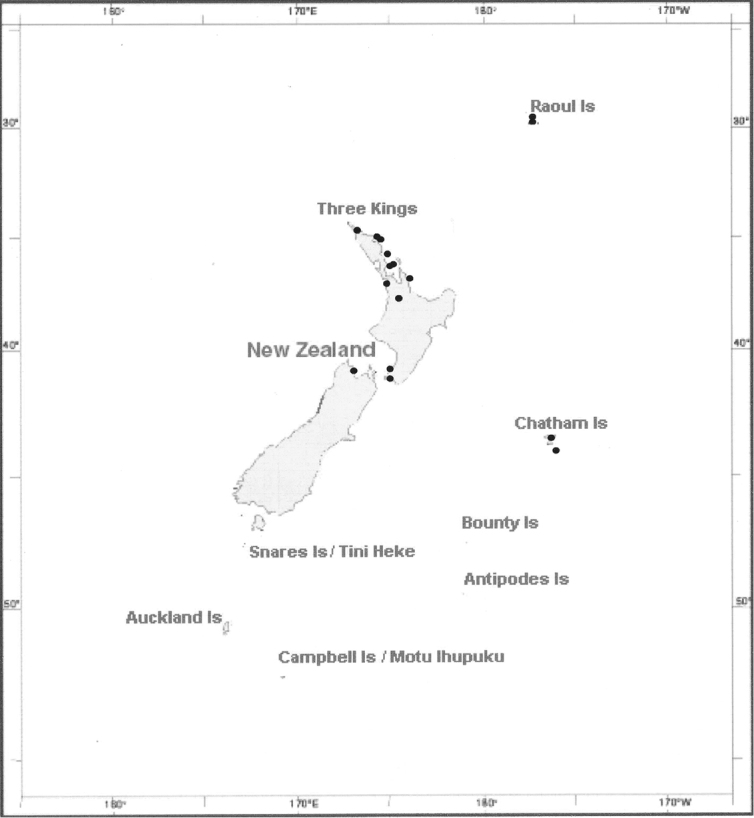
Indicative distribution. This map shows the known distribution of *Lejeunea hodgsoniana*.

#### Selected representative specimens examined.

**NEW ZEALAND, Kermadec Islands, North Meyer:** Kermadec Islands Nature Reserve, Kermadec Ecological Region and District, on exposed roots near trunk base of a cyclone toppled *Metrosideros kermadecensis*, 29°14'39.3"S, 177°52'34"W, ca. 60m, 12 May 2011, P. J. de Lange K670 (AK 325247); **Raoul Island:** Kermadec Islands Nature Reserve, Moumoukai Track, Moumoukai, Eastern side of summit, in wet forest on damp rock lying in deep shade of very large *Metrosideros kermadecensis* and *Melicytus* aff. *ramiflorus* trees, 29°16'0"S, 177°54'0"W, ca. 520m, 8 May 2009, P. J. de Lange K278, D C Havell (AK 313726); **Te Paki:** North Cape Scientific Reserve, Eastern tributary of the Nga Whenua Stream, Te Paki Ecological Region and District, growing amongst *Frullania pentapleura* on shaded serpentinite rocks lying in ephemeral stream bed, 34°24'12"S, 173°0'10"E, ca. 80m, 16 Nov 2010, P. J. de Lange 9918 (AK 323334); **Aorangi Island:** on ridge E of Puweto Valley, Poor Knights Ecological Region and District, epiphytic on trunk of *Cordyline obtecta*, ca. 35°28'40"S, 174°44'38"E,28 Aug 1984, J. E. Beever s.n. (AK 291340); **Hen and Chicken Group, Whatupuke Island:** Eastern Northland Ecological Region, Taranga Ecological District, epiphytic on horizontal trunk of large *Brachyglottis repanda*, ca. 35°53'20"S, 174°45'20"E, ca. 150m, 01 Jan 1982, J. E. Beever 8-59c (AK 291283); **Hen & Chicken Group, Lady Alice Island:** Eastern Northland Ecological Region, Taranga Ecological District, on trunks of living *Meryta* trees, ca. 35°53'36"S, 174°43'27"E, 07 Jan 1982, R. E. Beever, J. E. Beever 10-22a (AK291288); **Hen & Chicken Group, Lady Alice Island:** up Kereru Stream, coastal forest, epiphytic on trunk, sloping, 25 cm diameter lacebark, ca. 35°53’S, 174°43’E, 13 Dec 1981, J. E. Beever 8-12b (AK 291289); **Mokohinau Group, Fanal Island:** head of northern valley, on *Geniostoma*, at shrub/flax margin of forest, ca. 35°56'0"S, 175°9'0"E, 04 Jan 1984, E. K. Cameron 2711b (AK 291338); **The Noises:** Auckland Ecological Region, Inner Gulf Islands Ecological District, ca. 36°41'34"S, 174°58'28"E, Mar 1948,R. C. Lloyd 12 (AK 291347); **Auckland City:** Western Springs, Auckland Zoo grounds, Motions Creek, Tamaki Ecological District,, partially submerged on basalt rock, 36°51'47"S, 174°43'15"E, ca. 2 m, 04 Jan 2008, P. J. de Lange 7500 (AK 303709); **Port Waikato:** Eric Baker Memorial Scenic Reserve, Tainui Ecological Region, Raglan Ecological District, corticolous on kauri, 37°30'10"S, 174°47'40"E, ca. 40 m, 17 Feb 2009, P. J. de Lange 9160 (AK 313163); **Little Barrier Island:** Te Titoki Flat, 5 m E of bunkhouse, Coromandel Ecological Region, Little Barrier Ecological District, on bark of *Coprosma macrocarpa*, 36°13'18"S, 175°3'31"E, ca. 5 m, 29 Jan 1980, J. E. Braggins 80/1050, J. E. Beever (AK 291998); **Middle Island:** Coromandel Ecological District, Mercury Islands Ecological District, on *Melicytus ramiflorus* bark, 36°38'24"S, 175°51'42"E, ca. 60 m, 14 Dec 1983, E. K. Cameron 2504 (AK 291336); **Tuhua (Mayor Island):** Track to Devil’s Staircase, Mayor Ecological District, corticolous on puriri (*Vitex lucens*), 37°17'46"S, 176°15'46"E, ca. 180 m, 28 Jan 2012,P. J. de Lange 10600, T. J. de Lange, F. J. T. de Lange (AK 330653); **Hamilton:** Hamilton Basin, St Andrews, 9 Dover Road, Waikato Ecological Region, Hamilton Ecological District, corticolous on apple tree (*Malus x domestica*), 37°45'30"S, 175°15'23"E, ca. 35 m, 02 Jan 2012, P. J. de Lange 10550 (AK 330480); **Wellington:** Johnson Hill, Sounds-Wellington Ecological Region, Wellington Ecological District, on *Melicytus ramiflorus*, ca. 41°17’S, 174°44’E, 20 Apr 1969, B.G..Hamlin 1102 (WELT H000517); **Porirua:** Pauatahanui Inlet, north side, on *Melicytus ramiflorus* in small grove near shore, ca. 41°05'15"S, 174°53'12"E, 30 Apr 1969, B.G.Hamlin 1168 (WELT H000608); **Porirua:** Titahi Bay, Stuart Park, track from S end of bay, on trunk of *Melicytus ramiflorus*, 41°06'30"S, 174°49'48"E, ca. 10m, 30 Mar 2011, P. Beveridge LD-1 (WELT H012355); **Porirua:** Mitchell Stream, Spicer Botanical Park, on trunk of *Acacia dealbata* in open rank grass and weeds, S aspect, 41°09'38"S, 174°49'23"E, ca. 80m, 5 Oct 2012, P. Beveridge ME-1 (WELT H012566); **Mana Island:** lower part of Weta Valley, Cook Strait Ecological District, on lower trunk of *Coprosma repens* in lowland regenerating forest, 41°05'31"S, 174°46'52"E, ca. 15m, 10 Feb 2011, R.J..Lewington s.n. (CHR 608376); **Puponga:** Golden Bay, N.W.Nelson, on unsealed road linking Puponga and Farewell Spit, Northwest Nelson Ecological Region, West Wanganui Ecological District, growing on *Melicytus ramiflorus* bark in kanuka-dominant roadside scrub, 40°31'32"S, 172°44'34"E, ca. 25 m, 24 Feb 2012, G.G. Pritchard PFP-2 (WELT H012562); **Chatham Islands, Chatham Island:** Nikau Bush Scenic Reserve, Chathams Ecological Region and District, corticolous on *Melicytus chathamicus*, 43°46'0"S, 176°34'0"W, ca. 60 m, 28 Jun 2007, P. J. de Lange CH1021 (AK 301084); **Chatham Islands,Pitt Island:** stream above Canister Cove, on tree root above stream, ca. 44°20'04"S, 176°13'40"W, 05 Jan 1970, B.G. Hamlin 1102 (WELT H008409).

**Table 1.** Table of Locations – representative collections of *Lejeunea hodgsoniana*.

Latitude, Longtitude	Altitude	Location	Substrate	Herbarium
29°14'39"S, 177°52'34"W	ca. 60 m	Kermadec Islands northern group, North Meyer	Exposed roots of toppled *Metrosideros kermadecensis*	AK 325247
29°16'0"S, 177°54'0"W	ca. 520 m	Kermadec Islands, Raoul Island, Moumoukai	Wet forest on damp rock lying in deep shade	AK 313726
34°24'12"S, 173°0'10"E	ca. 80 m	Te Paki, North Cape, Eastern tributary of the Nga Whenua Stream	Shaded, serpentinite rocks lying in ephemeral stream bed	AK 323334
35°28'40"S, 174°44'38"E		Poor Knights, Aorangi Island, on ridge E of Puweto Valley	Epiphytic on trunk of *Cordyline obtecta*	AK 291340
35°53'20"S, 174°45'20"E	ca. 150 m	Hen and Chicken Group, Whatupuke Island	Epiphytic on horizontal trunk of large *Brachyglottis repanda*	AK 291283
35°53'36"S, 174°43'27"E		Hen & Chicken Group, Lady Alice Island	Trunks of living *Meryta trees*	AK 291288
35°53'S, 174°43’E		Hen & Chicken Group, Lady Alice Island, up Kereru Stream	Trunk, sloping, 25 cm diameter lacebark	AK 291289
35°56'0"S, 175°9'0"E		Mokohinau Group, Fanal Island, head of northern valley	On *Geniostoma*, *at shrub*/flax margin of forest	AK 291338
36°41'34"S, 174°58'28"E		Inner Gulf Islands Ecological District, The Noises		AK 291347
36°51'47"S, 174°43'15"E	ca. 2 m	Auckland City, Auckland Zoo grounds, Motions Creek	Partially submerged on basalt rock	AK 303709
37°30'10"S, 174°47'40"E	ca. 40 m	Port Waikato, Eric Baker Memorial Scenic Reserve	Corticolous on *Agathis australis*	AK 313163
36°13'18"S, 175°3'31"E	ca. 5 m	Little Barrier Island, Te Titoki Flat	On bark of *Coprosma macrocarpa*	AK 291998
36°38'24"S, 175°51'42"E	ca. 60 m	Mercury Islands Ecological District, Middle Island	On *Melicytus ramiflorus bark*	AK 291336
37°17'46"S, 176°15'46"E	ca. 180 m	Mayor Island, Track to Devil’s Staircase	Corticolous on *Vitex lucens*	AK 330653
37°45'30"S, 175°15'23"E	ca. 35 m	Hamilton City, St Andrews, 9 Dover Road	Corticolous on *Malus x domestica*)	AK 330480
41°17'S, 174°44'E		Wellington, Johnsons Hill	On *Melicytus ramiflorus*	WELT H000517
41°05'15"S, 174°53'12"E		Porirua, Pauatahanui Inlet, north side	On *Melicytus ramiflorus* *in small grove near* shore	WELT H000608
41°06'30"S, 174°49'48"E	ca. 10m	Porirua, Titahi Bay, Stuart Park, track from S end of bay	On trunk of *Melicytus ramiflorus*	WELT H012355
41°06'33"S, 174°49'43"E	ca. 15m	Porirua, Titahi Bay, Stuart Park, track to cliff edge at S end of bay	On trunk and branches of *Melicytus ramiflorus* *in coastal thicket*	WELT H012563
41°09'38"S, 174°49'23"E	ca. 80m	Porirua, Mitchell Stream, Spicer Botanical Park	On trunk of *Acacia dealbata* *in open rank g*rass and weeds	WELT H012566
41°05'31"S, 174°46'52"E	ca. 15m	Mana Island, lower part of Weta Valley	Trunk of *Coprosma repens* *in lowland reg* enerating forest	CHR 608376
40°31'32"S, 172°44'34"E	ca. 25 m	Puponga, Golden Bay, N.W.Nelson	On *Melicytus ramiflorus* *bark in kanuka-domi*nant scrub	WELT 012562
43°46'0"S, 176°34'0"W	ca. 60 m	Chatham Islands, Nikau Bush Scenic Reserve	Corticolous on *Melicytus chathamicus*	AK 301084
44°20'04"S, 176°13'40"W		Pitt Island, stream above Canister Cove	On tree root above stream	WELT H008409

#### Herbarium links for specimens examined.

New Zealand Virtual Herbarium NZVH www.virtualherbarium.org.nz provides links to the leading New Zealand herbaria.

The Australian Virtual Museum AVH http://avh.chah.org.au/ provides links to the leading Australian herbaria.

The direct links:

for WELT is: http://collections.tepapa.govt.nz/advancedsearch.aspx?CollectionGroup=NE this provides details of bryophyte collections although no images of the specimens cited above;

for the Allan Herbarium (CHR) is: http://nzflora.landcareresearch.co.nz/default.aspx?NavControl=search&selected=CollectionSearch;

for the bryophyte collection of the Field Museum of Natural History, Chicago (F) is: http://emuweb.fieldmuseum.org/botany/search_bryo.php

#### Recognition.

*Lejeunea hodgsoniana* is a distinctive species that can be recognized by a number of features that are unique among southern temperate Australasian *Lejeunea*: 1) the habit of shoots, with relatively large leaf-lobes closely appressed to the substrate is characteristic; 2) the multicellular, triangular first lobule tooth having a base up to four cells broad, is unique among Australasian species; 3) the elliptic-ovate, deeply divided underleaves with lobes capped (typically) by a single pointed cell are also unusual, occurring in no other *Lejeunea* from New Zealand; 4) the pentacarinate perianth with dorsal carina reduced or absent is also unusual, but not unique. The triangular, multicellular first lobule tooth, is not unique to *Lejeunea hodgsoniana* but is shared by at least two other *Lejeunea* species, *Lejeunea bidentula* Herzog and *Lejeunea kodamae* Ikegami & Inoue. However, *Lejeunea hodgsoniana* differs from both in details of the lobule and underleaf, and in the overall size of the plants. The lobule second tooth in *Lejeunea hodgsoniana* is never well developed, at best it is a broad, low and triangular with a weakly obtuse apex. Both *Lejeunea bidentula* and *Lejeunea kodamae* have a prominent, readily identifiable second lobule tooth, which is triangular in both species and has an acute to obtuse apex (Asthana and Saxena 2011). The underleaves of *Lejeunea bidentula* are shallowly bifid and broadly ovate, and those of *Lejeunea kodamae* are squat, almost rotund but for the sinus, in contrast to those borne by *Lejeunea hodgsoniana*. Both *Lejeunea bidentula* and *Lejeunea kodamae*, with shoots 0.7–0.9 mm wide, are smaller plants than *Lejeunea hodgsoniana* whose shoots frequently attain widths of 1.5 mm.

**Key to species with multicellular, triangular first lobule tooth in SE Asia and Australasia**

**Table:** 

1	Underleaves ovate, 5 × stem diameter, sinus to 0.3, broadly V-shaped, underleaf lobes not capped by prominent single cell. Gynoecia usually with two subfloral innovations, perianth pentacarinate, not dorso-ventrally flattened. Rostrum to seven cells	*Lejeunea bidentula*
–	Underleaves rotund to elliptic-ovate, 2–3 × stem diameter, sinus to 0.6, narrowly V-shaped, underleaf lobes capped by a prominent single cell. Gynoecia usually with a single subfloral innovation. Perianth pentacarinate, dorso-ventrally flattened or not. Rostrum to four cells	2
2	Shoots to 0.9 mm wide. First lobule tooth to two cells broad at base. Squat, rotund underleaves. Perianth not compressed, obovate. Rostrum to four cells	*Lejeunea kodamae*
–	Shoots to 1.5 mm wide. First lobule tooth to four cells broad at base. Elliptic-ovate underleaves. Perianth compressed, obcordate. Rostrum to three cells	*Lejeunea hodgsoniana*

#### Conservation.

*Lejeunea hodgsoniana* is widely distributed in coastal and lowland habitats in northern and southern parts of the North Island. It is abundant in mahoe dominated forests on the mainland and many offshore islands, including large areas within the conservation estate such as Hauturu and the Poor Knights Islands. The species occupies a wide range of forest habitats associated with high light environments, including forest edges, riparian vegetation, successional forest, and floodplain scrub. Within these vegetation types *Lejeunea hodgsoniana* can be found in highly disturbed remnants, as well as original stands, for instance coastal forests at Bream Tail, Northland. As a result, we consider this species to be Not Threatened according to the New Zealand Threat Classification System (Townsend et al. 2008).

#### Variation.

Throughout its range, there appears to be little variation from the range of variability expressed in the type material. In contrast to the usual weakly bulging leaf lobe cells, those in the sample on stream basalt from Motion Creek in Auckland, AK 303709, are moderately bulging giving a moderately crenulated margin to the lobes. Variation otherwise is in the size and shape of the perianth before sporophyte enlargement. The perianths in a sample from the Spicer Botanical Park in Porirua, WELT H012566, lacked the usual distinctly obcordate profile with the rostrum borne in a depression between the rounded apices of the lateral carinae. Instead, the apex of the perianth is truncate with rounded lateral carina apices. The rostrum is longer than usual at ca. 60 µm and the apical depression absent or almost so. Perianths in the samples AK 313726 from the summit of Raoul Island in the Kermadec Islands and AK 291289 from Lady Alice Island have similar truncate apices with a small or absent depression and, in the Kermadec sample, were smaller than usual at 350 µm high × 200 µm wide.

## Supplementary Material

XML Treatment for Lejeunea hodgsoniana
